# The relationship between professional identity and academic burnout among college students majoring in physical education a chain-mediated effect

**DOI:** 10.3389/fpsyg.2025.1618909

**Published:** 2025-09-15

**Authors:** Tianyi Wang, Yong Jiang

**Affiliations:** School of Physical Education, Liaoning Normal University, Dalian, China

**Keywords:** professional identity, academic burnout, physical education learning interest, achievement motivation, chain mediation model

## Abstract

Previous studies have shown that college students’ professional identity can negatively predict the phenomenon of academic burnout. However, college students majoring in physical education have many differences from other college students in terms of the content and form of learning, and there are fewer related studies, mostly at the level of the employment situation, and very few of them have been conducted on the subjective level of college students majoring in physical education. In addition, we tried to prove the chain mediating role of sports learning interest and achievement motivation in the relationship between professional identity and learning burnout of sports major college students. The respondents in the study, consisted of college students enrolled in physical education majors in the region of China. A total of 551 college students participated, including 383 males and 168 females. The study subjects were all undergraduate college students majoring in physical education. They completed the professional identity scale for undergraduates majoring in physical education, the physical education learning interest scale for undergraduates, the achievement motivation scale, and the learning burnout scale for undergraduates. The results found that there was a significant negative relationship between professional identity and burnout in the group of undergraduates majoring in physical education, and verified the mediating roles of physical education learning interest and achievement motivation. The findings also confirmed the existence of chain mediation among the four variables.

## 1 Introduction

In recent years, student burnout among university students has emerged as one of the significant challenges facing global higher education. According to the National College Health Assessment (NCHA) in the United States, 85% of college students have felt overwhelmed by academic pressure at some point during the academic year. Similarly, European studies have also indicated that 45%–71% of university students report symptoms of burnout, which manifest as emotional depression, decreased academic engagement, and even tendencies towards depression and dropout (Dopmeijer, 2021). In China, a meta-analysis further confirms that the overall prevalence of academic burnout among university students is 40.8%, with male students, those from non-key universities, and students in engineering and science disciplines demonstrating more pronounced levels of burnout (Sun and Liu, 2018).

Turhan et al. (2022) define academic burnout as a comprehensive psychological state in which students experience emotional decline, maladaptive behavior, and reduced sense of achievement due to prolonged academic pressure or lack of interest. Although the National Mental Health Act of the United States does not specifically address academic burnout, its policy direction that promotes the establishment of mental health service systems in higher education institutions has provided institutional support for alleviating student burnout.

Research on academic burnout has predominantly focused on the general population of university students, particularly in fields such as medicine, engineering, or comprehensive universities (Chen, 2023; Gong, 2023; Wang and Zhang, 2023; Liu and Jiang, 2024). However, students in sports-related disciplines have long been marginalized in mainstream research due to the unique nature of their curriculum structure, training load, and evaluation systems. Although a multidisciplinary study in Germany has indicated that the psychological state of students in sports and health sciences differs significantly from that of students in information technology and engineering regarding learning burnout and engagement, similar comparative studies investigating the psychological mechanisms within the field of sports remain scarce (Nils et al., 2025).

Based on the theoretical and policy background mentioned above, this study focuses on the group of university students majoring in sports, aiming to explore the intrinsic mechanism of the relationship between their professional identity and academic burnout. Furthermore, it seeks to examine the potential chain mediating effects of physical education learning interest and achievement motivation in this relationship.

## 2 Theory and hypothesis

### 2.1 The uniqueness of professional identity and academic burnout among college students majoring in physical education

The unique trajectory of physical education university students in terms of professional identity and academic burnout can be summarized as a “pre-given- delayed” model shaped by the dual logic of “body-institution,” which fundamentally differs from the “academic-post-mortem” path of liberal arts and sciences students.

The professional identity of sports students is anchored by competitive grades or technical standards before admission, and the basis of identity is the physical qualifications granted externally, rather than the academic community identity that needs to be won “later” through continuous knowledge absorption and output of arts and sciences. A longitudinal study by Moazami-Goodarzi et al. (2020) involving 391 elite Finnish high school athletes corroborated this difference: the competitive level prior to enrollment significantly predicted athletic identity, but had no significant effect on academic identity; the latter could only be gradually constructed in accordance with subsequent academic performance. Thus, student-athletes enter university with a considerable amount of inherent physical capital, and the university curriculum primarily serves to institutionalize the confirmation of this existing capital; in contrast, students in the humanities and sciences must begin accumulating knowledge capital from scratch, leading to the simultaneous occurrence of academic pressure and the construction of identity.

Another aspect of this original logic is the delayed activation of psychological exhaustion. The early specialized training undertaken by sports students during childhood faces diminishing marginal returns on physical capital in university, while institutionalized theoretical courses abruptly intrude into the training time-space, resulting in the “delayed detonation” of psychological exhaustion that was initially postponed. A qualitative study by Marangoni et al. (2023) involving five NCAA athletes experiencing high levels of burnout indicated that when academic tasks encroach upon training time, the “value stagnation” of physical capital, combined with academic load, ultimately triggers burnout. In contrast, the knowledge capital and academic pressure of students in the liberal arts and sciences accumulate in synchrony, with the path of their burnout resonating in harmony with the course progress. There is no niche burnout mechanism for sports students characterized by the “devaluation of past capital and the intrusion of current tasks.”

### 2.2 The impact of professional identity on academic burnout

Between professional identity and academic burnout, research has indicated that professional identity can significantly negatively predict the phenomenon of academic burnout (Jiang and Tian, 2018). Professional identity serves as the first psychological defense for sports students against high-pressure training and academic demands, with subjects that require a high level of professionalism imposing strong expectations on students’ sense of professional identity (Ju and Zhu, 2023). Henning defines it as “a subjective feeling of maintaining a balance between the inner self and professionalism,” emphasizing the dynamic coupling of emotional investment and cognitive belief in the interaction between individuals and their profession (Deng et al., 2020). In sports disciplines where action precision, competitive rules, and theoretical depth intertwine, the tighter this coupling, the more students can transform “I must practice” into “I am willing to practice,” thereby injecting sustained momentum into their subsequent learning behaviors.

However, the mere emotional affiliation is not sufficient to explain the reduction of burnout. Self-determination theory indicates that the protective effect of professional identity is primarily attributed to its simultaneous activation of three fundamental psychological needs: autonomy, competence, and relatedness (Ramos et al., 2025). When sports students internalize professional values as “I choose,” their need for autonomy is fulfilled; when technical evaluations and theoretical achievements receive recognition, their need for competence is met; and when team training and competition reinforce peer belonging, their need for relatedness is satisfied. These three needs collectively weaken the core dimensions of learning burnout, such as emotional exhaustion, cynicism, and diminished sense of achievement.

Therefore, we propose Hypothesis 1: Professional identity of sports university students negatively predicts academic burnout.

### 2.3 The mediating role of physical education learning interest

The concept of interest has a profound historical background in the field of psychology, traceable to the early pioneers of modern psychology, Herbart and Wendt (1835) who argued that there exists a close relationship between interest and learning (Schiefele, 1991). Interest can aid students in more accurately identifying and understanding the learning object, thereby promoting meaningful learning. Physical education learning interest refers to a psychological tendency of learners characterized by pleasurable emotions, the development of sports cognition, exploration, and sustained participation in sports (Cai et al., 2022).

Previous studies have suggested that there is a significant positive correlation between professional identity and learning interest (Hu et al., 2024; Khang and Hui, 2024). The enhancement of professional identity can strengthen students’ intrinsic motivation and self-efficacy in relation to their field of study, thereby stimulating and maintaining their interest in learning.

The theory of interest development suggests that an individual’s interest evolves from transient situational interest to stable individual interest (Hidi and Renninger, 2019). This process requires both emotional stimulation from external stimuli and a deep recognition of the value of the field. For university students majoring in sports, the self-construction of the identity of a “sports person” serves as this value anchor: the more they identify with their chosen major, the more they can transform the immediate pleasure derived from classes and training into a long-term passion for the discipline of sports, thus completing the psychological transition from being “attracted by activities” to “devoting oneself to sports as a career.”

Professional identity is precisely the “psychological fuel” driving this leap. Research shows that students with a high level of professional identity not only affirm the academic value of sports on a cognitive level but also experience a stronger sense of belonging and pride on an emotional level, and invest more time and energy in behavioral terms (Glandorf et al., 2024). This comprehensive engagement not only directly reduces dimensions of burnout such as emotional exhaustion and cynicism but also provides the necessary sense of self-efficacy and achievement feedback for the sustained development of interest.

Physical education learning interest serves as a crucial transition point from situational interest to individual interest, playing an intermediary role that connects the two: on one hand, it inherits the value affirmation and emotional energy gained from professional identification, allowing students to consistently obtain positive experiences during skill practice and theoretical study; on the other hand, it deepens the cognitive processing of sports through repeated positive experiences, ultimately solidifying into a stable individual interest, thus providing long-term relief from the exhaustion caused by learning pressure (Lin and Zhu, 2022).

Based on this, we propose Hypothesis 2: Physical education learning interest serves as a partial mediating role between professional identity and academic burnout among university students majoring in sports.

### 2.4 The mediating role of achievement motivation

Within the framework of goal achievement theory, the achievement motivation of college students can be further distinguished into two core pathways: “mastery orientation” and “performance orientation.” The former emphasizes enhancing self-ability and mastering the task itself, while the latter focuses on showcasing ability and gaining external evaluation (Urdan and Kaplan, 2020). Regardless of the orientation, achievement motivation consistently serves as an intrinsic driving force for the college student population, promoting the comprehensive growth of students.

Early literature has pointed out that individuals exhibit two distinct types of intrinsic motivational tendencies when faced with competition: the motivation to achieve success and the motivation to avoid failure (Atkinson, 1964). This indicates that the “approach-avoidance” dimension in achievement goal theory aligns perfectly with the “pursuit of success-avoidance of failure” framework found in traditional achievement motivation research, thus forming a theoretical resonance across these fields.

Research suggests that there is a significant positive correlation between professional identity and achievement motivation, making it one of the factors predictive of achievement motivation (Xu et al., 2023). Within the framework of achievement goal theory, this positive relationship can be understood as follows: when college students strongly identify with their chosen major, they are more likely to establish mastery-oriented goals, thereby enhancing their achievement motivation. Additionally, it has been pointed out that higher levels of achievement motivation can effectively alleviate academic burnout, while academic burnout can diminish students’ achievement motivation, leading to a reduction in their investment and effort in academics (Song, 2024).

The introduction of achievement goal theory can be further explained as follows: mastery goal orientation enhances task engagement and self-regulation, thereby reducing emotional exhaustion and depersonalization, which in turn mitigates learning burnout; conversely, performance goal orientation, when accompanied by a high avoidance tendency, may exacerbate learning burnout, subsequently undermining achievement motivation (Ariani, 2022).

From previous studies, it is understood that achievement motivation may be positively predicted by professional identity and negatively predicted by academic burnout. The question arises whether this conclusion can still be validated among the population of sports major university students.

Thus, Hypothesis 3 is proposed: Achievement motivation partially mediates the relationship between professional identity and academic burnout among sports major university students.

### 2.5 The chain mediation between physical education learning interest and achievement motivation

Although the aforementioned analysis has initially indicated that physical education learning interest and achievement motivation may independently mediate the relationship between professional identity and academic burnout among sports majors, this study further hypothesizes that the two do not simply operate in a parallel manner; rather, there is likely a chain mediation effect.

The independent mediating role of physical education learning interest and achievement motivation provides a logical basis for exploring their potential chained relationships. From a theoretical perspective, the dynamic connection between interest and motivation deeply aligns with the core essence of self-determination theory: an individual’s sustained engagement in a specific domain often begins with an emotional awakening triggered by interest, which then internalizes into a stable motivational system, ultimately shaping their behavioral outcomes. The research conducted by Steinmayr et al. (2019) (involving 345 students from high academic level schools in Germany) provides theoretical support for this. The study examined the impact of student motivation on academic performance, revealing that students’ self-concept of ability has a strong explanatory power for academic achievement. This finding indirectly supports the notion that professional identity may influence learning outcomes (such as academic burnout) through the mediating roles of learning interest and achievement motivation. It is particularly crucial that this research also reveals that learning interest is an important component of achievement motivation itself—it not only directly enhances academic performance but also indirectly promotes the level of achievement motivation by enhancing students’ intrinsic motivation and self-efficacy.

Hu’s research on the levels of learning interest has further deepened our understanding of the mechanisms by which interest operates (Hu, 2022). He categorizes learning interest into four progressive levels: intuitive interest, operational interest, causal interest, and theoretical interest, and particularly emphasizes the importance of fostering exploratory interest (i.e., causal interest and theoretical interest) in talent cultivation. This deeper level of interest serves as the core intrinsic motivation driving students to engage in scientific inquiry and self-directed learning, effectively stimulating their proactive involvement in related activities. Anderman’s analysis and research on achievement motivation theory clearly indicates that learning interest is one of the key antecedent variables in shaping achievement motivation (Anderman, 2020). It effectively stimulates students’ intrinsic motivation, prompting them to set more challenging achievement goals and to exert sustained effort towards them.

Focusing on the group of university students majoring in sports, this study hypothesizes that their professional identity may influence academic burnout, potentially following a causal chain of “professional identity → physical education learning interest → achievement motivation → academic burnout.”

Professional identity serves as the foundational source, providing individuals with value anchors and a sense of meaning in their engagement in the sports field. Physical education learning interest acts as a key driver, which, based on professional identity, leads to emotional investment and cognitive focus in sports learning (particularly high-level inquiry interest). This dual-driven approach, combining emotional appeal and cognitive value, effectively activates and enhances achievement motivation. As a behavioral engine, the activated achievement motivation (manifested through the pursuit of mastery, goal setting, and the desire for success) guides goal-directed behaviors such as setting objectives, increasing effort levels, and the perseverance to overcome difficulties, ultimately buffering or alleviating feelings of academic burnout.

In summary, this chain mediation mechanism not only aligns with the dynamic development pattern of psychological variables (identity → interest → motivation → behavioral outcomes) but also closely adapts to the unique training model of sports majors that emphasizes the deep integration of practice and theory.

**Figure 1:**
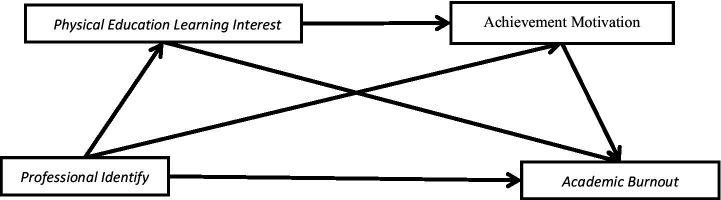
A hypothetical model of the effect of professional identity on academic burnout among college students majoring in physical education.

Therefore, this study proposes Hypothesis 4: physical education learning interest and achievement motivation play a chain mediating role in the relationship between professional identity and academic burnout among university students majoring in sports. Based on previous theories and studies, the hypotheses proposed for the relationship between the variables of this study are shown in Figure 1.

## 3 Materials and methods

### 3.1 Participants and procedures

The subjects of this survey are currently enrolled university students majoring in sports. The questionnaire was completed with the assistance of physical education teachers and college counselors, and respondents completed it anonymously. All individuals filling out the questionnaire participated as unpaid volunteers. At the beginning of the questionnaire, respondents were informed that their information would be kept confidential and used solely for this research.

A total of 605 questionnaires were distributed, of which 54 invalid questionnaires were excluded due to insufficient time for answering (26), incorrect attitude towards answering (28) and other problems, and 551 valid questionnaires were recovered, with the effective recovery rate of the questionnaires being 91.07%. Among them, 383 were male college students and 168 were female college students, and the grade distribution was from freshman to senior.

This study was approved by the Ethics Committee of Liaoning Normal University (Ethical Review Number: LL2024074). Prior to the study, permission for questionnaire testing and informed consent from the students themselves were obtained. All students participated in the survey voluntarily.

### 3.2 Analytic strategy

This study used spss27.0 and process V4.0 tools to conduct common method bias test, correlation analysis, regression analysis and mediation effect analysis on the data. Bootstrap mediation effect test was conducted for physical education learning interest and achievement motivation under the condition that the significance level of confidence interval was 95%, and the mediation effect was significant when the confidence interval did not contain zero.

Common method bias refers to the artificial covariation between the predictor variables and the validity variables caused by the same data sources or raters, the same measurement environment, the program context, and the characteristics of the program itself. This artificial covariation is a form of systematic error that can seriously confound the results of a study and potentially mislead the conclusions. The 60 questions in the scale were analyzed by Harman’s one-way test. The results of the test showed that the eigen roots of six factors were >1, and the explained variance of the first factor was 30.696%, which was lower than the critical criterion of 40%. This indicates that there is no obvious common method bias.

### 3.3 Professional identify

The Professional Identity Scale, known as the Professional Identity Scale for College Students Majoring in Physical Education. The scale was compiled by Liao (2011). The scale selects 20 questions and is divided into five dimensions: professional emotional identity, professional identity cognitive identity, professional behavioral identity, professional matching identity, and professional value identity.

During the scoring process, we calculate the sum and average of the scores for each item to obtain data for analysis. The higher the score the stronger the professional identity of sports. The scale is scored on a five-point scale, and the Cronbach’s alpha coefficient is 0.966, indicating that the reliability of the questionnaire is good.

### 3.4 Physical education learning interest

Physical education learning interest Scale, the full name is “Physical Education Learning Interest Scale for College Students.” The scale was compiled by Gu and Xie (2012). The scale selects 20 questions and is divided into five dimensions: positivity, negativity, skill learning, after-school activities, and sports concern.

During the scoring process, we calculate the sum and average of the scores for each item to obtain data for analysis. The higher the score the stronger the physical education learning interest. The scale was scored on a five-point scale, and the Cronbach’s alpha coefficient was 0.955, indicating that the reliability of the questionnaire was good.

### 3.5 Achievement motivation

The Achievement Motivation Scale (AMS) was jointly developed by Gjesme and Nygard in 1970, compiled by Ye Renmin and the Norwegian scholar Hegtve in 1988, and finally revised and completed in 1992 (Ye and KuntA, 1992). The scale consists of 30 questions, including two parts to measure the motivation to pursue success and avoid failure. The score of achievement motivation (M) consists of the score of the part of pursuing success (Ms) minus the score of the part of avoiding failure (Maf), and the higher the score is, the stronger the motivation for achievement is.

During the scoring process, we calculate the sum and average of the scores for each item to obtain data for analysis. Eight questions were selected from the scale to study the achievement motivation of the university students who majored in physical education in the school. The scale was scored on a four-point scale, the average value is taken for the measurement of Cronbach and the Cronbach’s alpha coefficient was 0.840, indicating that the reliability of the questionnaire was good.

### 3.6 Academic burnout

The Learning Burnout Scale for College Students was compiled by Lian et al. (2005), including three dimensions of low mood, inappropriate behavior, and low sense of accomplishment, and the higher the score is, the stronger the learning burnout situation is Lian et al. (2005). Twelve questions in the scale were selected to study the learning burnout situation of college students majoring in physical education.

The scale was scored on a five-point scale, the average value is taken for the measurement of Cronbach and the Cronbach’s alpha coefficient was 0.886, indicating that the reliability of the questionnaire was good.

## 4 Results

### 4.1 Descriptive statistical analyses between variables

The results of the independent samples *t*-test in Table 1 show that only in the dimension of “professional identity” did the difference between male students (*M* = 3.865, SD = 0.934) and female students (*M* = 4.082, SD = 0.598) reach statistical significance (*t* = −3.265, *p* = 0.006); no significant gender differences were found for the remaining variables - physical education learning interest, motivation to achieve, and academic burnout (*p* > 0.05).

**Table 1:** Sample size demographic information and independent sample *t*-test.

Dependent variable	Gender (*N* = 551)	*M* ± SD	*t*	*p*
Professional identify	Male (383)	3.865 ± 0.934	−3.265	0.006
	Female (168)	4.082 ± 0.598		
Physical education learning interest	Male (383)	3.517 ± 0.807	−0.678	0.498
	Female (168)	3.566 ± 0.731		
Achievement motivation	Male (383)	3.008 ± 0.663	0.211	0.833
	Female (168)	2.995 ± 0.620		
Academic burnout	Male (383)	2.333 ± 0.797	0.606	0.545
	Female (168)	2.289 ± 0.724		

The one-way ANOVA in Table 2 shows that there were no statistically significant differences between grades for any of the four variables of major identity, physical education learning interest, achievement motivation, and academic burnout from freshman to senior year (*p* > 0.05 corresponding to all *F*-values).

**Table 2:** Sample size, demographic information, and one-way ANOVA.

Dependent variable	Grade (*N* = 551)	*M* ± SD	*F*	*p*
Professional identify	1 (131)	3.952 ± 0.833	1.953	*p*>0.05
	2 (139)	3.785 ± 0.943		
	3 (142)	3.980 ± 0.817		
	4 (139)	4.009 ± 0.795		
Physical education learning interest	1 (131)	3.542 ± 0.784	0.618	*p*>0.05
	2 (139)	3.455 ± 0.832		
	3 (142)	3.573 ± 0.786		
	4 (139)	3.555 ± 0.735		
Achievement motivation	1 (131)	3.053 ± 0.667	2.000	*p*>0.05
	2 (139)	2.910 ± 0.598		
	3 (142)	3.081 ± 0.655		
	4 (139)	2.972 ± 0.669		
Academic burnout	1 (131)	2.319 ± 0.766	1.843	*p*>0.05
	2 (139)	2.427 ± 0.834		
	3 (142)	2.210 ± 0.731		
	4 (139)	2.324 ± 0.759		

1, first-year university student; 2, second-year university student; 3, Third-year university student; 4, fourth-year university student, the same below.

### 4.2 Descriptive statistical analyses between variables

As shown in Table 3, Pearson correlation analysis was conducted for professional identity, interest in physical education learning, achievement motivation, and academic burnout. The results show that there are significant correlations among the four variables, with significant positive correlations among professional identity, physical education learning interest and achievement motivation, and significant negative correlations between all three and academic burnout. It provides support for further construction of structural equation modeling.

**Table 3:** Pearson correlation coefficient.

	Professional identify	Physical education learning interest	Achievement motivation	Academic burnout
Professional identify	1			
Physical education learning interest	0.374**	1		
Achievement motivation	0.314**	0.353**	1	
Academic burnout	−0.357**	−0.457**	−0.344**	1

^**^p<0.01.

### 4.3 Testing the mediating of social support and extroversion

As shown in Table 4, the chain mediation effect was tested using Model 6 in the SPSS PROCESS macro program developed by Hayes. Gender and grade level were used as control variables to test the chain mediation model in this study.

**Table 4:** Regression analysis between variables.

Equation of regression		Overall fit index			Significance of regression coefficient		
Result variable	Variable of prediction	*R*	*R* ^2^	*F*	*β*	*t*	*p*
M1	Gender	0.374	0.140	29.694	−0.015	−0.379	0.705
	Grade				0.004	0.110	0.912
	X				0.376***	9.392	0.000
M2	Gender	0.407	0.166	27.151	−0.047	−1.194	0.233
	Grade				−0.027	−0.696	0.487
	X				0.219***	5.153	0.000
	M1				0.273***	6.476	0.000
Y	Gender	0.524	0.274	41.182	0.002	0.065	0.948
	Grade				−0.015	−0.420	0.674
	X				−0.180***	−4.427	0.000
	M1				−0.329***	−8.061	0.000
	M2				−0.172***	−4.297	0.000

X, professional identify; M1, physical education learning interest; M2, achievement motivation; Y, academic burnout. *p<0.05; **p<0.01; ***p < 0.001.

The regression results in Table 4 indicate that professional identity significantly and positively predicts physical education learning interest (*β* = 0.376, *p* < 0.001) and achievement motivation (*β* = 0.219, *p* < 0.001), while physical education learning interest also significantly and positively predicts achievement motivation (*β* = 0.273, *p* < 0.001). Moreover, professional identity significantly and negatively predicts academic burnout (*β* = −0.180, *p* < 0.001), and both physical education learning interest and achievement motivation significantly and negatively predict academic burnout (*β* = −0.329, *p* < 0.001; *β* = −0.172, *p* < 0.001). Gender and grade level showed no significant effects in any of the equations (*p* > 0.05).

**Figure 2:**
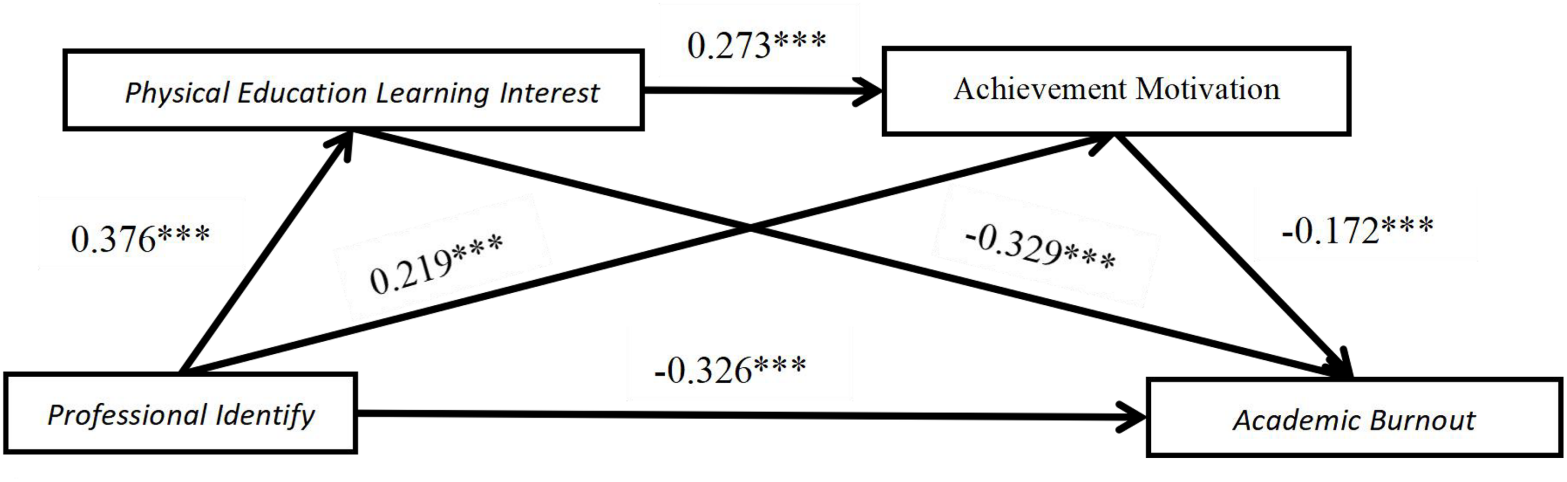
A model of the mediating role of physical education learning interest and achievement motivation in the relationship between professional identity and academic burnout. ***p < 0.001, significant regression coefficient.

Figure 2 visually illustrates this chain mediation model, clearly presenting the directional relationships and path significance among the four variables. It graphically explains how professional identity influences academic burnout through both direct and indirect pathways (including the independent mediating roles of physical education learning interest and achievement motivation, as well as their chain mediating role).

The results of the study (Table 5) showed that, first, there was a significant negative correlation between professional identity and academic burnout among college students majoring in physical education, supporting Hypothesis 1.

**Table 5:** Proportion of the mediating effect.

Influence path	Effect size	Boot SE	95% confidence interval		Proportion
			BootLLCI	BootULCI	
Total effect	−0.326	0.037	−0.398	−0.254	100%
Direct effect	−0.164	0.037	−0.236	−0.091	50.12%
Total indirect effect	−0.163	0.027	−0.218	−0.113	49.84%
Path1	−0.113	0.021	−0.156	−0.073	34.66%
Path2	−0.034	0.012	−0.061	−0.014	10.43%
Path3	−0.016	0.005	−0.026	−0.008	4.91%

Path 1: Professional identity → Physical education learning interest → Academic burnout; Path 2: Professional identity → Achievement motivation → Academic burnout; Path 3: Professional identity → Physical education learning interest → Achievement motivation → Academic burnout.

Second, professional identity has a significant indirect effect on academic burnout through physical education learning interest [*β* = −0.113, 95%CI = (−0.156, −0.073)], i.e., physical education learning interest plays a significant mediating role in the relationship between professional identity and academic burnout among physical education majors, supporting Hypothesis 2.

Third, professional identity has a significant indirect effect on academic burnout through achievement motivation [*β* = −0.034, 95%CI = (−0.061, −0.014)], i.e., achievement motivation plays a significant mediating role in the relationship between professional identity and academic burnout among physical education majors, supporting Hypothesis 3.

Fourth, professional identity has a significant indirect effect on academic burnout through the chain path of physical education learning interest → achievement motivation [*β* = −0.016, 95%CI = (−0.026, −0.008)], i.e., physical education learning interest and achievement motivation play a significant chain mediating role in the relationship between professional identity and academic burnout among physical education majors, supporting Hypothesis 4.

## 5 Discussion

As expected, in a population of college students majoring in physical education, physical education learning interest and achievement motivation played the roles of separate and chain mediators in the relationship between professional identity and academic burnout. This result contributes to a more comprehensive understanding of the intrinsic mechanisms of the effects of academic burnout in the group of college students majoring in physical education, and provides a theoretical basis for improving the motivation and professional stability of this group.

### 5.1 Significant differences in professional identity by gender

The study found significant differences between male and female physical education majors in terms of professional identity, and this additional finding is well worth exploring.

From a socio-cultural perspective, the field of sports has long been given a strong masculine label, and social acceptance and expectations of male participation in sports majors are generally higher, and this implicit support from the external environment may have strengthened male students’ identification with sports majors (Zehntner et al., 2023).

At the level of professional practice, many courses in physical education majors are more compatible with males in terms of physiological functions, which makes it easier for them to gain a sense of achievement in their professional learning and thus strengthen their professional identity; at the same time, the difference in gender role perception may lead to female students being more prone to self-doubt about “whether they are suitable for it” in the study of physical education majors, and this kind of internal cognitive conflict will weaken their sense of professional belonging (Liu et al., 2023).

Parsing this gender difference can provide an important addition to understanding the mechanisms of professional identity formation among physical education students, as well as laying the foundation for the subsequent development of differentiated professional guidance strategies.

### 5.2 The relationship between professional identity and academic burnout among college students majoring in physical education

From the results of the study, professional identity is a negative predictor of academic burnout in a population of college students majoring in physical education, i.e., the stronger a student’s professional identity, the lower his or her level of academic burnout tends to be.

Although some studies have also found that professional identity negatively predicts academic burnout, most of these studies have focused on general college students or other groups, and there are fewer studies focusing on a specific population (Alghtany et al., 2024).

Specifically, when students have a high degree of recognition of their majors, they will naturally categorize themselves as members of the professional community (Hu and Cheung, 2024). This sense of belonging will push them to actively follow the norms and values of the professional field, actively engage in professional learning and strive to improve their own professional ability.

Students with a strong sense of professional identity have a clearer perception and higher value recognition of their majors, which makes them more motivated and enthusiastic in the learning process, and able to take the initiative to cope with the difficulties and challenges in their studies, which in turn reduces the emergence of burnout (Chen et al., 2023).

Synthesizing the relevant discussions and research results, it can be seen that professional identity can alleviate academic burnout to a certain extent and help students maintain a good learning state and psychological state.

### 5.3 The mediating role of physical education learning interest on professional identity and academic burnout among college students majoring in physical education

When students identify themselves with their majors, they often feel that the contents of their majors are highly compatible with their own interests, plans and values, and this sense of identification will naturally stimulate their interest in learning (Tan et al., 2024). Interest in learning itself is a powerful internal motivation, can effectively guide and maintain continuous learning behavior, when the interest in learning has been enhanced, students will be more willing to invest time and energy in the learning process, and it is easier to reap the benefits of fun and satisfaction, which is precisely the vivid embodiment of the concept of “Teaching for Fun” (Wong et al., 2020).

By devoting themselves to learning, students’ curiosity and desire for knowledge will be satisfied, and the sense of burnout will be reduced. Because a strong interest in learning can help them effectively relieve pressure and negative emotions, so that they always maintain a positive attitude and full of enthusiasm in learning. On the contrary, if students lack of professional identity, they may find the content of learning boring, and it is difficult to generate interest in learning, and then easy to fall into the state of learning burnout (Bergdahl et al., 2024).

Therefore, professional identity can effectively reduce learning burnout and help students better engage in their professional learning by enhancing their interest in learning.

### 5.4 The mediating role of achievement motivation on professional identity and academic burnout among college students majoring in physical education

Individual behavior and psychological state are driven by both intrinsic and extrinsic motivation (Wang et al., 2024). Professional identity can satisfy the basic psychological needs for autonomy, competence and sense of belonging, and thus stimulate intrinsic motivation.

Individuals who have a higher level of identification with their profession usually view the tasks and challenges of the field in a more positive light, and this attitude further strengthens their achievement motivation (Faihs et al., 2023). This attitude will further strengthen their achievement motivation, and the stimulated achievement motivation will drive individuals to be more active in learning and practicing, and strive to achieve professional goals and accomplishments. At the same time, the enhancement of achievement motivation will make individuals more focused and engaged in learning, and more willing to actively cope with all kinds of learning tasks, thus effectively reducing the emergence of learning burnout.

Thus, professional identity, by reinforcing achievement motivation, enables people to maintain a high level of motivation and drive throughout the learning process, which both reduces the risk of learning burnout and provides strong support for professional development and learning effectiveness.

### 5.5 The chain mediating role of physical education learning interest and achievement motivation on professional identity and academic burnout among college students majoring in physical education

The phenomenon of learning burnout among college students majoring in physical education is a multifactorial and complex problem that involves multiple psychological, educational and social dimensions. Studies have shown that learning burnout is common among college students majoring in physical education, and the degree is close to medium level (Barcza-Renner et al. 2024).

Physical education learning interest and achievement motivation build a logical chain between professional identity and learning burnout. Professional identity enhances physical education learning interest and achievement motivation, and eventually inhibits learning burnout.

This study not only helps to understand the dynamic changes in the psychological state of college students majoring in physical education, but also provides a theoretical basis and practical guidance for the curriculum design and teaching management of physical education, in order to promote the positive development of students in the process of physical education, reduce the occurrence of the phenomenon of learning burnout, and improve the quality of education and the learning experience of students (Lochbaum et al., 2022).

In order to reduce the learning burnout of college students majoring in physical education, we can start from three aspects: professional identity, physical education learning interest and achievement motivation.

First of all, enhancing the sense of professional identity is the key. Research shows that professional identity and learning burnout are significantly negatively correlated, that is, the higher the professional identity, the lower the degree of learning burnout (Yukhymenko-Lescroart, 2021). Therefore, by optimizing the curriculum, increasing practical teaching links and improving students’ sense of professional identity, learning burnout can be effectively reduced (Madigan et al., 2024).

Secondly, it is also crucial to enhance the physical education learning interest. According to research, physical education learning interest has a significant effect on learning burnout, and students with strong interest are more active in learning (Liu et al., 2024). Students’ physical education learning interest can be stimulated through diversified teaching methods and rich extracurricular activities.

Finally, cultivating achievement motivation is also an effective way to reduce learning burnout. Students with higher levels of achievement motivation are more motivated and confident in facing learning challenges (Zhang et al., 2024). Teachers should give students more positive feedback, help them set reasonable learning goals, and enhance their motivation to pursue success (Wang et al., 2025).

Through these comprehensive measures, the learning burnout of college students majoring in physical education can be effectively reduced and their overall development promoted.

## 6 Conclusion

In this study, we investigated the relationship between professional identity and academic burnout by targeting college students majoring in physical education, and confirmed that professional identity has a significant negative predictive effect on academic burnout. At the same time, we verified the independent mediating role of sports learning interest and achievement motivation in the relationship between the two, as well as the chain mediating effect of “professional identity → sports learning interest → achievement motivation → academic burnout” formed by the two, which reveals the internal mechanism behind this relationship.

This study fills in the gap of the research in this field in the group of college students majoring in physical education, deepens the understanding of the intrinsic relationship between professional identity and academic burnout, and provides important theoretical support and practical guidance for the alleviation of academic burnout among college students majoring in physical education in educational practice by enhancing professional identity, cultivating learning interest and strengthening achievement motivation.

## 7 Limitations and future directions

There are also some shortcomings in this study. First, due to the limitations of time, research funding and other objective factors, this study utilized a cross-sectional research design.

Although existing studies provide a solid theoretical foundation for this study, the results of this study can be enriched and expanded through further follow-up and empirical studies.

Second, the subjects of this study were all current college students majoring in physical education, although from different colleges and universities, there are still some geographical limitations. In future research, the influencing factors of learning burnout among college students majoring in physical education between different geographic regions can be further investigated.

Finally, learning burnout in this study was self-reported by participants through questionnaires. Specific study burnout influencing factors need to be further explored.

## Data Availability

The original contributions presented in the study are included in the article/Supplementary material, further inquiries can be directed to the corresponding author.
