# Supplementary material for: The relationship between professional identity and academic burnout among college students majoring in physical education a chain-mediated effect
Source: Front Psychol. 2025 Sep 15;16:1618909. doi: 10.3389/fpsyg.2025.1618909 (PMC12477245; doi:10.3389/fpsyg.2025.1618909)
Supplement: Supplementary file 2 [file Table_1.DOC]

**TABLE 1|Sample size demographic information and independent sample *t* test.**

| Dependent variable | *Gender（N*=551） | M±SD | t | p |
| --- | --- | --- | --- | --- |
| Professional Identify | Male（383） | 3.865±0.934 | -3.265 | 0.006 |
|  | Female（168） | 4.082±0.598 |  |  |
| Physical Education Learning Interest | Male（383） | 3.517±0.807 | -0.678 | 0.498 |
|  | Female（168） | 3.566±0.731 |  |  |
| Achievement Motivation | Male（383） | 3.008±0.663 | 0.211 | 0.833 |
|  | Female（168） | 2.995±0.620 |  |  |
| Academic Burnout | Male（383） | 2.333±0.797 | 0.606 | 0.545 |
|  | Female（168） | 2.289±0.724 |  |  |

**TABLE 2|**Sample size, demographic information and one-way ANOVA.

| Dependent variable | Grade（N=551） | M±SD | F | p |
| --- | --- | --- | --- | --- |
| Professional Identify | 1（131） | 3.952±0.833 | 1.953 | p＞0.05 |
|  | 2（139） | 3.785±0.943 |  |  |
|  | 3（142） | 3.980±0.817 |  |  |
|  | 4（139） | 4.009±0.795 |  |  |
| Physical Education Learning Interest | 1（131） | 3.542±0.784 | 0.618 | p＞0.05 |
|  | 2（139） | 3.455±0.832 |  |  |
|  | 3（142） | 3.573±0.786 |  |  |
|  | 4（139） | 3.555±0.735 |  |  |
| Achievement Motivation | 1（131） | 3.053±0.667 | 2.000 | p＞0.05 |
|  | 2（139） | 2.910±0.598 |  |  |
|  | 3（142） | 3.081±0.655 |  |  |
|  | 4（139） | 2.972±0.669 |  |  |
| Academic Burnout | 1（131） | 2.319±0.766 | 1.843 | p＞0.05 |
|  | 2（139） | 2.427±0.834 |  |  |
|  | 3（142） | 2.210±0.731 |  |  |
|  | 4（139） | 2.324±0.759 |  |  |

1,first-year university student；2,second-year university student；3,third-year university student；4,fourth-year university student,the same below.

**TABLE 3|**Pearson correlation coefficient.

|  | Professional Identify | Interest in physical education learning | Achievement Motivation | Academic Burnout |
| --- | --- | --- | --- | --- |
| Professional Identify | 1 |  |  |  |
| Physical Education Learning Interest | 0.374^**^ | 1 |  |  |
| Achievement Motivation | 0.314^**^ | 0.353^**^ | 1 |  |
| Academic Burnout | -0.357^**^ | -0.457^**^ | -0.344^**^ | 1 |

**：p＜0.01.

**TABLE 4|**Regression analysis between variables.

| **Equation of regression** | | **Overall fit index** | | | | **Significance of**  **regression coefficient** | | |
| --- | --- | --- | --- | --- | --- | --- | --- | --- |
| Result variable | Variable of prediction | R | R^2^ | F | | β | t | p |
| M1 | Gender | 0.374 | 0.140 | | 29.694 | -0.015 | -0.379 | 0.705 |
|  | Grade |  |  |  |  | 0.004 | 0.110 | 0.912 |
|  | X |  |  |  |  | 0.376*** | 9.392 | 0.000 |
| M2 | Gender | 0.407 | 0.166 | | 27.151 | -0.047 | -1.194 | 0.233 |
|  | Grade |  |  |  |  | -0.027 | -0.696 | 0.487 |
|  | X |  |  |  |  | 0.219*** | 5.153 | 0.000 |
|  | M1 |  |  |  |  | 0.273*** | 6.476 | 0.000 |
| Y | Gender | 0.524 | 0.274 | 41.182 | | 0.002 | 0.065 | 0.948 |
|  | Grade |  |  |  |  | -0.015 | -0.420 | 0.674 |
|  | X |  |  |  |  | -0.180*** | -4.427 | 0.000 |
|  | M1 |  |  |  |  | -0.329*** | -8.061 | 0.000 |
|  | M2 |  |  |  |  | -0.172*** | -4.297 | 0.000 |

X,Professional Identify;M1, Physical Education Learning Interest；M2,Achievement Motivation;Y,Academic Burnout.*: p＜0.05;**:p＜0.01; ***:p<0.001.

**TABLE 5|**Proportion of the mediating effect.

0.219****

-0.326***

*Professional Identify*

*Physical Education Learning Interest*

Achievement Motivation

*Academic Burnout*

**FIGURE 2** | A model of the mediating role of Physical Education Learning Interest and achievement motivation in the relationship between professional identity and academic burnout. *** p<0.001, Significant regression coefficient.

-0.329****

0.376****

-0.172****

0.273****

| **Influence path** | **Effect size** | **Boot SE** | **95% confidence interval** | | **Proportion** |
| --- | --- | --- | --- | --- | --- |
|  |  |  | **BootLLCI** | **BootULCI** |  |
| Total effect | -0.326 | 0.037 | -0.398 | -0.254 | 100% |
| Direct effect | -0.164 | 0.037 | -0.236 | -0.091 | 50.12% |
| Total indirect effect | -0.163 | 0.027 | -0.218 | -0.113 | 49.84% |
| Path1 | -0.113 | 0.021 | -0.156 | -0.073 | 34.66% |
| Path2 | -0.034 | 0.012 | -0.061 | -0.014 | 10.43% |
| Path3 | -0.016 | 0.005 | -0.026 | -0.008 | 4.91% |

Path 1: Professional identity → Physical education learning interest → Academic burnout；Path 2: Professional identity → Achievement motivation → Academic burnout；Path 3: Professional identity → Physical education learning interest → Achievement motivation → Academic burnout
